# Real-time and recollected ratings of pain, mood, and fatigue in older adults

**DOI:** 10.1097/PR9.0000000000001326

**Published:** 2025-08-20

**Authors:** Rachel L. M. Ho, Trey B. Warren, Jinhan Park, Young Seon Shin, Matthew Petersen, Yenisel Cruz-Almeida, Stephen A. Coombes

**Affiliations:** a Laboratory for Rehabilitative Neuroscience, Department of Applied Physiology and Kinesiology, University of Florida, Gainesville, FL, USA; b Department of Cardiology, College of Medicine, University of Florida, Gainesville, FL, USA; c Department of Community Dentistry, Pain Research and Intervention Center of Excellence, University of Florida, Gainesville, FL, USA; d Department of Biomedical Engineering, University of Florida, Gainesville, FL, USA

**Keywords:** Pain intensity variability, Ecological momentary assessment, Pain recall

## Abstract

**Introduction:**

Pain intensity variability refers to the fluctuations in pain intensity levels experienced by an individual over time. Technological advances have simplified the use of ecological momentary assessment (EMA) paradigms for researchers and participants allowing for easier measurement of pain intensity variability by measuring pain intensity multiple times a day over several days. In this study, we used EMA to investigate the relationship between variability in pain intensity, mood, and fatigue and accuracy of recall in older adults.

**Methods:**

Twenty-six cognitively normal participants aged 60 and above completed EMA 3 times daily over 14 days, rating their pain intensity, mood, and fatigue using a smartphone application. On the 15th day, participants were asked to recall their average levels of these variables.

**Results:**

We found that greater variability in pain intensity was significantly associated with poorer recall accuracy, even when controlling for peak pain, end pain, and the number of painful areas. However, no significant associations were found between variability and recall accuracy for mood or fatigue.

**Conclusion:**

These results suggest that the difficulties with recall accuracy are specific to pain intensity and do not extend to mood or fatigue, highlighting the importance of considering pain intensity variability in understanding and managing pain in older adults.

## Abstract 2

Associations between variability and discrepancy are unique to pain intensity in older adults and do not extend to mood and fatigue.

## 1. Introduction

Pain is highly prevalent among older adults, with some studies estimating that ∼53% of individuals over the age of 65 experience persistent pain.^37^ Although many studies have demonstrated that pain fluctuates over time^20,28,38^ and have examined the experience of pain along with its associated psychological variables,^6,21,32,46^ pain intensity variability has only recently begun to receive attention.^3,24,36^ Pain intensity variability refers to fluctuations in pain intensity over time, or intraindividual pain variability.^34^ Understanding pain intensity variability has important implications for interpreting pain that is reported by recall. Indeed, pain intensity is often measured at a single time point, relying on participants' recall of their average pain intensity over time (eg, the previous 2 weeks, 3 months).^8,22^ Pain diaries, and more recently, digital tools for ecological momentary assessments (EMAs) allow for greater temporal resolution when measuring pain intensity.^35,40^ Greater pain intensity variability is associated with poorer recall of pain,^16,28,38^ which is independent of sex,^20^ and is represented by a greater difference between the mean of the momentary ratings and the single recalled value. What remains unclear is whether the association between variability and recall is similar across different domains (eg, general mood or fatigue) or is unique to pain. Given the well-established associations between pain intensity, mood, and fatigue,^7,9,10,44^ in the current study we examine the association between variability and recall for pain intensity, mood, and fatigue.

When individuals recall an experience, they focus disproportionately on the most intense moments (peaks) and the final moment (end).^1,12,19,27^ Evidence in support of the peak-end rule comes from Redelmeier et al.^39^ who tracked pain levels every minute during colonoscopy procedures. Pain recall immediately after the procedure and 1 hour later was best predicted by the peak pain level and the pain experienced in the last minute of the procedure. However, Stone et al.^43^ found that the peak level of pain over the course of a week was not associated with recall. Methodological differences in the temporal resolution of the ratings may account for differences between studies. The impact of the peak-end rule on the recalled value may, therefore, be influenced by the duration and temporal resolution of the ratings. Whether peak-end scores contribute to the relationship between variability and recall for pain intensity, mood, and fatigue is not known.

In the current study, older adults completed pain, mood, and fatigue ratings 3 times a day, for 14 days using a customized smartphone application. We focused on cognitively normal older adults because pain is more prevalent as we age.^37^ If the association between variability and recall, and the peak-end rule, is generalizable, one would expect significant and similar associations across domains.

## 2. Methods

### 2.1. Participants and study design

A total of 26 participants completed this study. We utilized EMA data from the Understanding Pain Biology in Elders Across Time study (UPBEAT), a longitudinal study investigating pain-related brain changes as predictive factors of age-related mobility decline. Inclusion criteria included (1) being ≥60 years old, (2) fluent in written and spoken English, (3) having scored >29 on the Telephone Interview for Cognitive Status questionnaire, and (4) having access to the internet and a personal cellular device.

### 2.2. Baseline questionnaires

Participants completed a series of questionnaires including the following: (1) demographics questionnaire, (2) location of worst pain and pain duration, and (3) graded chronic pain scale (GCPS; intensity and disability subscales).

### 2.3. Ecological momentary assessment survey and schedule

**Figure 1.**
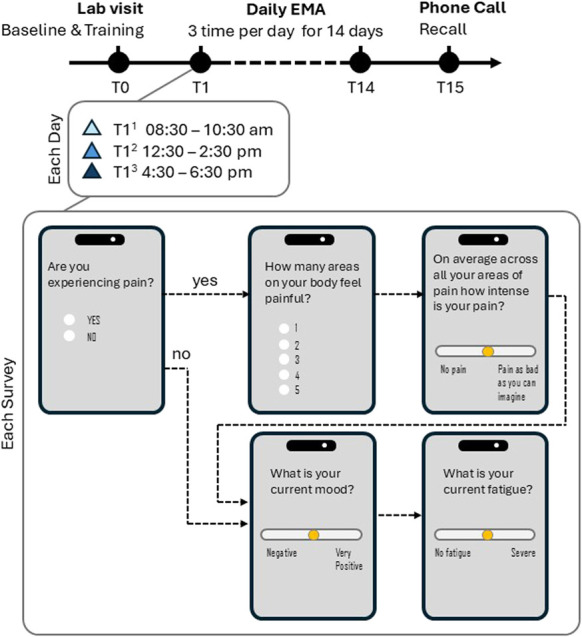
Associations between absolute discrepancy scores and pain intensity, mood, and fatigue variability: 3 correlations were used to measure the associations between variability and absolute discrepancy for pain intensity, mood, and fatigue. In (A–C), the *x* axis shows variability scores, and the *y* axis shows absolute discrepancy scores. Dots represent the data from each participant, and the black line represents a linear fit derived from simple linear regression. (A) Shows that greater pain intensity variability is significantly and positively associated with greater absolute discrepancy scores. (B and C) Show that the association between variability and absolute discrepancy score for mood and fatigue was not significant.

Figure 1 displays a flow diagram outlining the study protocol. Our EMA survey was composed of 3 main topics: (1) pain, (2) mood, and (3) fatigue (see “Each Survey” section at bottom of Fig. 1). To assess pain, participants were asked, “Are you currently experiencing pain?” and responded with either “yes” or “no.” If participants answered no, the survey moved to the questions of mood and fatigue. If participants answered yes, they received the following questions: “How many areas on your body feel painful?”, and “on average across all your areas of pain how intense is your pain?”. For the question regarding how many locations on the body feel painful, participants were allowed to choose between 1 and 5 locations. The question on pain intensity used a continuous slider visual analog scale (VAS), which allowed participants to slide the marker down to “no pain” or up to “pain as bad as you can imagine.” Although the participant could not see numbers, in the background the slider location corresponded to a number between 0 and 100. Questions about mood and fatigue also used a continuous slider VAS scale with mood being anchored by “negative” and “very positive” and fatigue being anchored by “no fatigue” and “severe.” No pain, negative mood, and no fatigue were associated with a score of 0, whereas pain as bad as you can imagine, very positive mood, and severe fatigue were associated with scores of 100.

Participants received 3 EMA surveys a day (see “Each Day” section of Fig. 1). The survey asked the same questions at each delivery. The first survey was delivered between the hours of 8:30 am and 10:30 am, the second between the hours of 12:30 and 2:30 pm, and the third between the hours of 4:30 and 6:30 pm. Participants were allowed to choose their preferred delivery time for the survey within each specified time range and were informed that they have an hour to complete each survey (eg, if they chose 9 am, they would have from 9 am until 10 am to complete the survey). Surveys took less than 5 minutes to complete. Surveys were delivered 3 times a day for 14 days total. Study staff helped participants download the application (mEMA-sense, ilumivu) and allowed participants ample time to practice with their device and ask questions (T0 in Fig. 1). Participants received a guidebook, which reminded them of days and time when they would receive surveys, provided troubleshooting instructions, and listed appropriate contact information if they had any issues or questions.

### 2.4. Variables of interest: absolute discrepancy scores, ecological momentary assessment variability, peak scores, end scores, and painful areas

We followed the same procedures outlined by Stone et al.^43^ to calculate variability measures and absolute discrepancy scores. Specifically, after the 14-day study period, study staff called participants on day 15 to ask them recall questions regarding pain intensity, mood, and fatigue (T15 in Fig. 1). Participants were told to expect a phone call on day 15 but not the questions they would be asked. We reminded them that they received 3 surveys per day for 14 days, each time rating their pain intensity. We then asked them to provide an average rating based on their recollected individual ratings (pain intensity recall). We repeated these steps for mood and fatigue to receive a single recalled value for mood and fatigue (mood recall/fatigue recall). The top of Figure 1 illustrates the protocol timeline. Time point zero (T0) is when participants attended the lab to download the mEMA-sense application and receive training. After this appointment, participants completed 14 days of surveys in their own environment (time points 1–14; T1–T14). At time point 15 (T15), participants received a phone call to ask them recall questions regarding pain intensity, mood, and fatigue.

**Figure 2.**
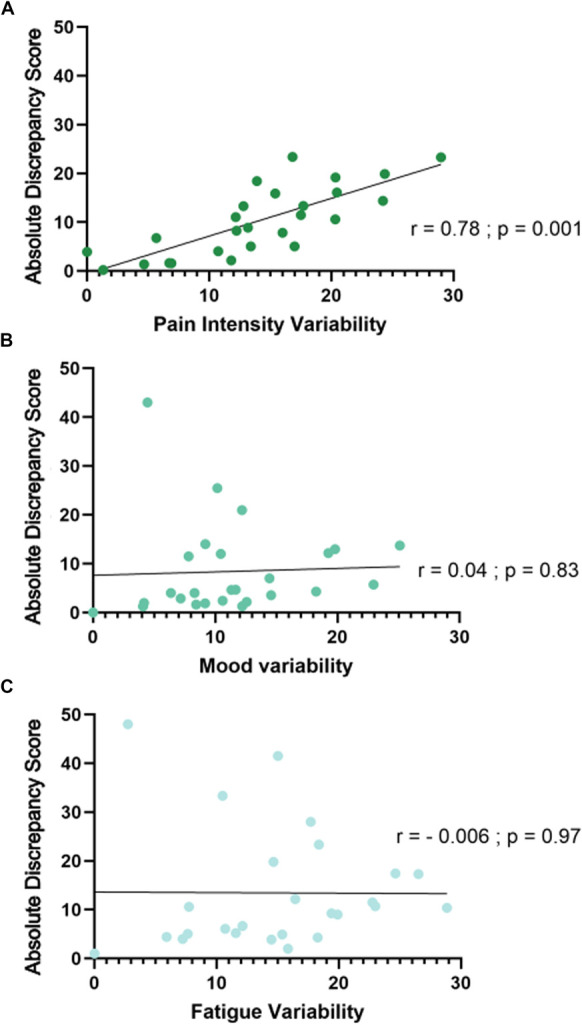
Study Protocol: Participant received 3 surveys a day for 14 days on their mobile device. The mobile application delivered a survey that asked questions about pain, mood, and fatigue (see “Each Day” section of Fig. 1). To access pain, participants were asked, “Are you currently experiencing pain?” and responded either “yes” or “no.” If participants answered no, the survey moved to the questions of mood and fatigue. If participants answered yes, they received the following questions: “How many areas on your body feel painful?”, and “on average across all your areas of pain how intense is your pain?”. For the question regarding how many locations on the body feel painful, participants were allowed to choose between 1 and 5 locations. The question on pain intensity used a continuous slider visual analog scale (VAS) ,which allowed participants to slide the marker down to “no pain” or up to “pain as bad as you can imagine.” Although the participant could not see numbers, in the background the slider location corresponded to a number between 0 and 100. Questions about mood and fatigue also used a continuous slider VAS scale with mood being anchored by “negative” and “very positive” and fatigue being anchored by “no fatigue” and “severe.” No pain, negative mood, and no fatigue were associated with a score of 0, whereas pain as bad as you can imagine, very positive mood, and severe fatigue were associated with scores of 100. The first survey was delivered between the hours of 8:30 am and 10:30 am, the second between the hours of 12:30 and 2:30 pm, and the third between the hours of 4:30 and 6:30 pm (see Each Day section of Fig. 1). Study staff helped participants download the application and allowed them ample time to practice with their devices and ask questions (Lab visit T0 in Fig. 1). Participants left the lab and filled out surveys on their own 3 times a day for 14 days (T1 to T13 in Fig. 1). After the 14-day study period, study staff called participants on day 15 to ask them recall questions regarding pain intensity, mood, and fatigue (T15 in Fig. 1). EMA, ecological momentary assessment.

After receiving recall scores for pain intensity, mood, and fatigue, we then calculated absolute discrepancy scores. Pain intensity absolute discrepancy scores (see equation below) were measured by first calculating each participants' pain intensity EMA average, which corresponded to the average pain intensity across all survey instances. Pain intensity recall was then subtracted from the pain intensity EMA average, culminating in a pain intensity absolute discrepancy score for each subject. We chose to calculate absolute scores to avoid positive and negative values canceling each other out. In addition, our primary focus was to determine accuracy of recall irrespective of direction. Finally, previous studies suggest that individuals tend to overestimate their pain.^13,14,26^ The same steps were repeated for calculating absolute mood and fatigue discrepancy scores.

Absolute discrepancy score calculation:

|Pain intensity recall−Pain intensity EMA average|

To evaluate how absolute discrepancy scores relate to variability, we calculated each participants' pain intensity standard deviation across all survey instances. The same step was done to calculate mood and fatigue variability. Standard deviations serve as a measure of variability across the 2 weeks and are labeled here as pain intensity variability, mood variability, and fatigue variability. Pain intensity peak scores were calculated for each individual by finding the maximum pain intensity rating across all surveys. The same steps were completed to calculate peak mood and fatigue scores. For calculating pain intensity end scores, an average was taken across all the surveys completed on the last day. For example, if a participant completed 3 surveys on the last day, their rating for pain intensity was averaged from the 3 surveys. This was repeated to calculate end scores for mood and fatigue.

Finally, to investigate the influence of the variability of number of painful areas on pain intensity absolute discrepancy scores and pain intensity variability, we calculated the standard deviation of number of pain locations for each participant across all the surveys. Moving forward, this variable will be called painful areas variability. We also calculated average number of pain locations to characterize our sample.

### 2.5. Data analysis and statistics

The primary goal of the study was to examine how pain intensity variability is associated with pain intensity absolute discrepancy scores in older adults, and whether the relationship between variability and absolute discrepancy scores is also present for measures of mood and fatigue. To investigate this goal, we ran 3 separate Pearson Product correlations to measure the association between absolute discrepancy scores and variability. A significant positive correlation would mean that higher variability was associated with worse recall. Next, to test the possibility that peak scores, end scores, or painful areas variability helps explain the relationship between pain intensity variability and absolute discrepancy scores, we ran partial correlations where peak scores, end scores, or painful areas variability were covaried. For mood and fatigue, we performed the same steps to investigate if peak scores and end scores help explain the relationship between variability and absolute discrepancy scores by running partial correlations where peak scores or end scores were covaried. Consistent with the approach taken by Stone et al.^43^ to further explore characteristics of our sample, significant findings were further explored by testing for significant differences between 2 groups (high variability vs low variability). Groups were identified using a k-means split with a setting of 2 clusters on the variability measure. Once delineating our 2 groups into a high and low variability group, we then ran an independent samples *t* test to determine whether absolute discrepancy scores were significantly different between groups.

Finally, we conducted Pearson Product correlation analyses to examine the relationships among several variables: (1) pain intensity recall vs pain intensity EMA average, (2) mood recall vs mood EMA average, (3) fatigue recall vs fatigue EMA average, (4) pain intensity absolute discrepancy scores vs mood absolute discrepancy scores, (5) pain intensity absolute discrepancy scores vs fatigue absolute discrepancy scores, and (6) mood absolute discrepancy scores vs fatigue absolute discrepancy scores.

## 3. Results

### 3.1. Participant characteristics

Table 1 displays the demographic information for participants, of which 73% were female, 84% white, and 100% non-Hispanic. The age of participants ranged from 65 to 84 years, with an average age of 71.5 (SD: 5.3) years. The most common location for worst pain was low back at 30.8% and pain duration for worst location was 10.5 (SD: 12.3) years. Participants had an average GCPS intensity score of 55.5 (SD: 21.3) and an average GCPS disability score of 37.5 (SD: 25.6). For the direction of calculated discrepancy scores, 80.7% of our participants overestimated (positive discrepancy scores) their pain intensity and mood, whereas 85.6% overestimated their fatigue.

**Table 1:** Characteristics of participants (N = 26).

Characteristic	No. of participants	%	Average	SD
Age (y)			71.5	5.3
Sex				
Female	19	73		
Male	7	27		
Race				
Black	3	11		
White	22	84		
American Indian or Alaskan Native	1	3		
Ethnicity				
Hispanic	0	0		
Non-Hispanic	26	100		
Worst pain location				
Hand	2	7.7		
Shoulder	4	15.4		
Low back	8	30.8		
Hip	2	7.7		
Knee	7	26.9		
Foot	3	11.5		
Pain duration (y)			10.5	12.3

Across all participants, across 14 days there were 1,092 surveys delivered. A total of 959 were completed (87%). The average compliance rate was 86% (SD: 10.0). Table 2 summarizes the average and standard deviation of all our variables of interest. Ecological momentary assessment averages for pain intensity, mood, fatigue, and number of locations were calculated by taking all the answers across all participants and measuring the average and standard deviation. Peak and end averages were calculated by taking the peak and end score from each participant and measuring the average and standard deviation across all 26 participants. In general, participants had mild to moderate amounts of pain (mean = 27.9 on a 0–100 scale), high mood (mean = 73.8 on a 0–100 scale), low fatigue (mean = 28.3 on a 0–100 scale), and the average number of pain locations was 1.4 locations.

**Table 2:** Averages and standard deviations for main variables of interest across all participants.

Variable	Average	SD	Range
Momentary pain intensity	27.9	28.3	0–100
Pain intensity recall score	36.3	25.1	0–82
Pain intensity absolute discrepancy score	10.3	7.0	0.22–24
Peak pain intensity	58.2	29.0	2–100
End pain intensity	29.9	29.3	0–74
Momentary mood	73.8	22.2	0–100
Mood recall score	83.1	27.7	60–100
Mood absolute discrepancy score	8.4	9.6	0–25
Peak mood	91.4	11.1	56–100
End mood	76.4	22.1	11–100
Momentary fatigue	28.3	26.8	0–100
Fatigue recall score	38.8	27.6	0–90
Fatigue absolute discrepancy score	13.5	12.3	1–48
Peak fatigue	65.0	26.6	1–100
End fatigue	28.9	27.1	1–94
No. of locations	1.40	1.2	0–5

SD, standard deviation.

### 3.2. Associations between pain intensity variability, mood variability, fatigue variability, and absolute discrepancy scores

Figure 2 shows the results from the 3 correlations used to measure the associations between variability and absolute discrepancy for pain intensity, mood, and fatigue. In Figure 2A–C, the *x* axis shows variability scores, and the *y* axis shows absolute discrepancy scores. Dots represent the data from each participant, and the black line represents a linear fit derived from simple linear regression. Figure 2A shows that greater pain intensity variability is significantly and positively associated with a greater absolute discrepancy score (r = 0.78; *P* = 0.001). We ran 3 separate partial correlations to control for the potential confounds of peak, end, and painful areas variability scores. Significant positive correlations were found even when controlling for peak pain intensity (r = 0.72; *P* < 0.001), end pain intensity (r = 0.78; *P* < 0.001), and painful areas variability (r = 0.72; *P* < 0.001). Compared to the zero-order correlation (r = 0.78), these results indicate that peak pain intensity and painful areas variability had a negligible influence, whereas end pain intensity had no influence on the relationship between pain intensity variability and absolute discrepancy scores.

Figure 2B shows that the association between variability and absolute discrepancy score for mood was not significant (r = 0.04; *P* = 0.83), which remained nonsignificant when controlling for peak (r = −0.17; *P* = 0.58) and end scores (r = −0.15; *P* = 0.458). The relationship between variability and absolute discrepancy scores for fatigue was also not significant (Fig. 2C: r = −0.006; *P* = 0.97), irrespective of whether peak and end were controlled for (peak: r = −0.12; *P* = 0.58; end: r = 0.02; *P* = 0.94).

Since pain intensity variability was the only measure to have a significant relationship with absolute discrepancy scores, we performed a k-means split using pain intensity variability. The split produced 2 clusters: a low (N = 14) and high (N = 12) variability group. Average pain intensity absolute discrepancy score for each group was significantly different, t(1, 26) = −1.814, *P* = 0.041. The high variability group had a significantly higher average absolute discrepancy score (M = 45.6, SD = 25.7) compared to the low variability group (M = 28.4, SD = 22.5).

We conducted 6 Pearson correlations analyses to examine relationships among key study variables, with results presented in Table 3. Two correlations were significant before and after false discovery rate correction^5^: mood recall vs mood EMA average and fatigue recall vs fatigue EMA average. These results indicate that mood recall is associated with the EMA average mood, and fatigue recall is associated with the EMA average fatigue.

**Table 3:** Correlation results.

Correlation analysis		r-value	*P*	FDR corrected *P*
Pain recall	Pain intensity EMA average	0.3423	0.087	0.174
Mood recall	Mood EMA average	0.7470	0.00001	0.00003
Fatigue recall	Fatigue EMA average	0.8331	0.0000001	0.000006
Pain intensity absolute discrepancy score	Mood absolute discrepancy score	0.1676	0.168	0.252
Pain intensity absolute discrepancy score	Fatigue absolute discrepancy score	−0.0475	0.852	0.860
Mood absolute discrepancy score	Fatigue absolute discrepancy score	−0.0340	0.8688	0.860

EMA, ecological momentary assessment; FDR, false discovery rate.

## 4. Discussion

The present study used an EMA paradigm to examine whether variability in pain intensity, mood, and fatigue ratings over time are associated with recall accuracy in older adults. We report 2 novel findings. First, increased variability in pain intensity was associated with poorer recall, even when controlling for peak, end, and multisite pain. Second, no significant relationship was found between variability and recall for mood or fatigue. These results indicate that difficulty with recall is specific to pain intensity and is not generalizable to mood and fatigue.

Pain intensity variability may be an important phenotype in individuals with chronic pain.^17,29,45,47^ For instance, when subgrouping individuals with sickle cell disease,^2^ chronic low back pain,^45^ and fibromyalgia^3^ based on levels of pain intensity variability, between group differences were also evident in the intensity of pain, medication use, physical function, fatigue, and mood. Greater pain intensity variability was associated with worse scores on psychosocial measures. This growing body of evidence suggests that measuring and understanding pain intensity variability has important implications on the race to improve personalized pain medicine and for better understanding the complex nature of pain.^17,18,29,34,47^

Increased variability was associated with poorer recall for pain intensity but not for mood or fatigue. Our findings cannot be attributed to attenuated variability in mood and fatigue scores, given that all variables were scored on a 0 to 100 scale, and the standard deviation and range for each measure were similar (see Table 2). Similarly, the mean and standard deviation for absolute discrepancy scores were within similar ranges across factors. As such, it does not appear to be inherently easier to recall pain, mood, or fatigue. Rather, for mood and fatigue, recall is influenced by factors other than variability.

Recalling mood is easier when an individual's current mood is congruent with the mood they are being asked to recall.^15,30^ Hence, the mood individuals are in when asked to recall past moods may influence their recollections. This may explain the absent association between mood variability and absolute discrepancy scores, as one's mood on recollection day may disproportionally alter the recall score. In addition, we acknowledge that our questions regarding mood were general (eg, reported along a spectrum of good or bad). It is plausible that more detailed questions (Beck Depression Inventory II,^4^ Patient Health Questionnaire-9)^42^ regarding mood could have produced different results, with the caveat that this appraoch would be significantly more time consuming for participants to answer 3 times a day. Finally, our sample of participants reported high mood (more positive) in both momentary mood and recall of mood. Research on the “well-being paradox” suggests that subjective well-being increases and remains stable into the mid-70s.^23^ The average age of our sample was 71.5 years old, and our sample displayed relatively positive mood during the 2-week period. These results may reflect the well-being paradox. Results in Table 2 suggest that there were comparable amounts of variability between pain intensity and mood, but overall high levels of mood may have influenced our results.

Fatigue variability is often studied in the context of symptom management and has been assessed in individuals with chronic fatigue syndrome and fibromyalgia.^3,41^ To our knowledge, only 1 paper has investigated the relationship between fatigue variability and absolute discrepancy scores.^41^ Higher variability in fatigue ratings were less accurate at recall of fatigue. In the current study, we found no association between fatigue variability and absolute discrepancy scores. Key differences between the study by Sohl et al.^41^ and this study relate to cohort diagnosis, age, sample size, and EMA methods. Sohl et al. collected data from 53 individuals with chronic fatigue syndrome who were aged 18–60 years old, whereas we collected data from 26 community-dwelling older adults with an average age of 71.5 years, with varying amounts of pain. Although Sohl et al.^41^ did not report the average age of their participants, their inclusion criteria would indicate that their cohort was significantly younger than ours. In addition, the authors wanted to capture diurnal patterns of fatigue in their participants, so they administered their survey 6 times a day for 21 days. It is plausible that although fatigue is a symptom associated with pain in older adults,^11,25^ younger individuals with chronic fatigue syndrome experience a different type of fatigue that is difficult to capture fully when asked to recollect over a week. Indeed, our participants reported an average momentary fatigue of 28.3 and standard deviation of 26.8, whereas Sohl et al. reported an average momentary fatigue of 49.3 and standard deviation of 17.3 (4.93 ± 1.73 on their 0–10 scale) across the 3 weeks, thus reporting higher levels of fatigue with less variability than our cohort. Finally, it is possible that the increased number of surveys a day compared to our methods helped to capture the association between fatigue variability and recall absolute discrepancy in this patient population. Finally, our sample of participants reported low amounts of fatigue in both momentary fatigue and recall of fatigue. Although physiological changes associated with aging can increase fatigue,^31,33,48^ our sample displayed relatively low amounts of fatigue across the 2 week period. Table 2 suggests that there were comparable amounts of variability between pain intensity and fatigue, but overall low levels of fatigue may have influenced our results.

We note several limitations to the current study. First, we did not control or analyze for differences in sex and our sample size was small with a lack of racial diversity among participants. The sample predominantly consisted of white individuals (84%), and this imbalance may limit the generalizability of our findings to other racial groups. In addition, although participants reported the number of locations where they experienced pain, they did not report the location itself. Although some locations of the body may be more sensitive to pain than others (eg, the hand vs the gastrocnemius muscle), we did not track nor analyze these data in the study. Future studies should consider expanding the sample to increase generalizability, stratifying data based on pain location or conducting separate analyses for different body regions to better understand the effect of location of pain on pain intensity variability.

## 5. Conclusion

This study investigated the relationship between pain intensity variability, mood, fatigue, and recall accuracy in older adults. The results indicate that greater variability in pain intensity was significantly associated with poorer recall accuracy, even after accounting for factors such as peak pain, end pain, and the number of painful areas. No significant associations were found between variability and recall accuracy for mood or fatigue. These findings suggest that the difficulties with recall accuracy are specific to pain intensity and do not extend to other subjective experiences like mood and fatigue. Clinicians and researchers should be mindful of the potential impact of pain intensity fluctuations on an individual's ability to accurately recall their pain experience, as this may have implications for pain assessment and treatment planning.

## Disclosures

The authors have no conflicts of interest to declare. S.A.C. is cofounder and manager of Neuroimaging Solutions, LLC.
